# Suspected Metformin-induced Cobalamin Deficiency Mimicking Thrombotic Thrombocytopenic Purpura

**DOI:** 10.7759/cureus.6921

**Published:** 2020-02-08

**Authors:** Syed Ather Hussain, Mohammad Ammad Ud Din, L. K. Teja Boppana, Ankita Kapoor, Saad Jamshed

**Affiliations:** 1 Internal Medicine, Rochester General Hospital, Rochester, USA; 2 Hematology/Oncology, Rochester Regional Health, Rochester, USA

**Keywords:** metformin, cobalamin deficiency, pseudo-ttp, thrombotic microangiopathy, b12 deficiency, micd

## Abstract

Thrombotic thrombocytopenic purpura (TTP) can often be life threatening and requires timely diagnosis and prompt initiation of plasmapharesis. Cobalamin deficiency can closely mimic TTP and distinguishing between the two diseases can prove to be a diagnostic challenge. Previously, cobalamin-related pseudo-TTP has been associated with pernicious anemia, dietary insufficiency and hereditary disorders of cobalamin activation. Here in, we discuss the first case of suspected metformin-induced cobalamin deficiency causing pseudo-TTP. Our patient was a 36-year-old female with type 2 diabetes mellitus on metformin for eight years who presented with hemolytic anemia, thrombocytopenia, schistocytes and mild acute renal failure. The initial impression was TTP; however, further workup revealed very low serum cobalamin levels and elevated methylmalonic acid levels. Apart from metformin use, no other cause of cobalamin deficiency was identified. We recommended upper gastrointestinal endoscopy to definitively rule out pernicious anemia.

## Introduction

Thrombotic microangiopathies are a heterogeneous group of diseases, which can be congenital or acquired, have an acute or gradual onset, and can manifest early or late in life [1]. Thrombotic thrombocytopenic purpura (TTP) is one of these diseases with an incidence of four cases per 1,000,000 in the United States [2]. TTP can often be life threatening and requires timely diagnosis and prompt initiation of plasmapharesis. The term "pseudo-TTP" has been coined for cobalamin deficiency presenting with thrombocytopenia, hemolytic anemia and schistocytosis. In the past, pseudo-TTP secondary to cobalamin deficiency has been linked to pernicious anemia, decreased dietary intake and autosomal recessive disorders of cobalamin activation [2]. To the best of our knowledge, we discuss the first case of suspected metformin-induced cobalamin deficiency (MICD) causing pseudo-TTP. Further we discuss the important aspects of the workup and management in light of the current available literature.

## Case presentation

A 36-year-old non-vegan African American female, with a past medical history of childhood obesity, diabetes mellitus type 2 (DM2) for eight years and iron-deficiency anemia (IDA) for one year, presented to us with complaints of progressively worsening fatigue for six weeks, followed by upper and lower extremity cramping, joint stiffness and hand paresthesia over a five-week period. She reported a history of regular heavy menstrual bleeding requiring up to 10 pads per day. She was diagnosed with IDA a year ago after which she was started on oral iron supplementation. She denied any nausea, vomiting, melena, appetite or weight changes. She had no prior history of fibroids or prior blood transfusions.

In the past couple of days, she had worsening fatigue and had a presyncopal episode, which prompted her to visit her primary care physician (PCP). The PCP ordered blood work, which showed severe anemia with a hematocrit of 10.8% and hemoglobin 3.7 g/dL with no other abnormalities. Therefore, she was asked to visit the hospital for further management.

Her family history was significant for breast cancer in the maternal aunt. She had had five children and no history of miscarriages. She was a former smoker, drank alcohol occasionally and denied any drug use. Of note, she was taking metformin 1,000 mg twice daily for DM2 for eight years.

**Figure 1:**
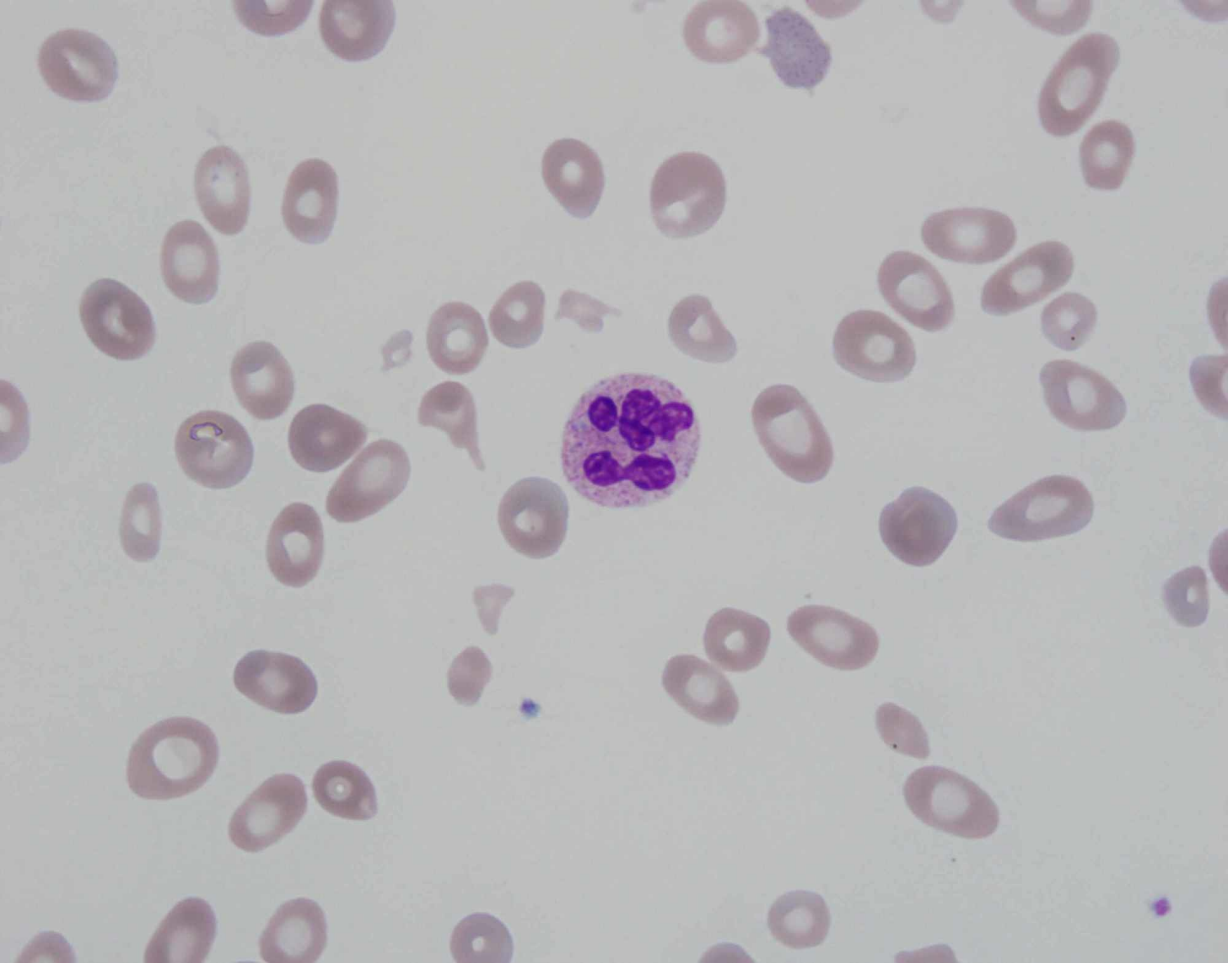
Peripheral blood smear showing hypersegmented neutrophil and schistocytes. Image at 1,000x magnification

**Figure 2:**
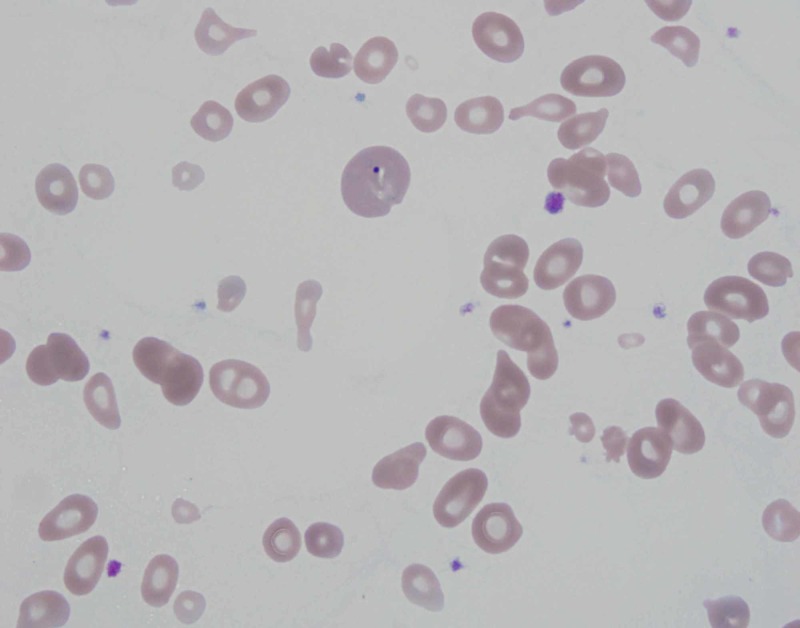
Peripheral blood smear showing poikilocytosis, anisocytosis and teardrop cells. Image at 1,000x magnification

On presentation to the emergency department, she had a heart rate of 110 beats/min, blood pressure 129/68 mmHg, respiratory rate 23 breaths/min, temperature 37°C and O_2_ saturation 99% on room air. Electrocardiogram showed sinus tachycardia. Repeat lab work revealed pancytopenia with white cell count 2.4 x 10^3^/μL, hemoglobin 3.7 g/dL, hematocrit 11% and platelet count 94 x 10^3^/μL. Peripheral blood smear showed schistocytes (Figure 1), anisocytosis (Figure 2), macrocytosis and microcytosis. Complete metabolic panel was notable for blood urea nitrogen 22 mg/dL, creatinine 1.2 mg/dL (baseline 0.6-0.8 mg/dL), total bilirubin 1.4 mg/dL, direct bilirubin 0.3 mg/dL, indirect bilirubin 1.1 mg/dL, aspartate aminotransferase 113 U/L and alanine aminotransferase 50 U/L. Coagulation studies showed an international normalized ratio 1.0, activated partial thromboplastin time 23.6 seconds and fibrinogen 312 mg/dL. 

Gynecology was consulted regarding heavy menses, and the patient was started on medroxyprogesterone 20 mg p.o. twice daily which helped to stop the bleeding; pelvic ultrasound was ordered which showed a normal endometrial stripe of 3.76 mm. Thyroid-stimulating hormone was also checked which came back normal at 1.74 uIU/mL.

Hematology was consulted regarding concern for TTP, and the patient was transferred to the medical intensive care unit for the management of anemia and suspected TTP. Further workup revealed lactate dehydrogenase (LDH) 4,320 U/L, haptoglobin <1 mg/dL, reticulocyte count percentage 1.7% and reticulocyte count number 19.3 x 10^3^/μL. For a very high LDH, the number of schistocytes seen on the peripheral blood smear was thought to be low and that raised suspicion for cobalamin deficiency. Consequently, cobalamin level was ordered which was found to be 91 pg/mL and methylmalonic acid level was 31 nmol/mL. Serum folate was 16.6 ng/mL. Iron studies showed iron 88 mcg/dL, total iron-binding capacity 334 mcg/dL, iron saturation 26% and ferritin 79 mcg/L. Parvovirus B19 antibody IgM was negative. Rheumatologic workup included antinuclear antibody, rheumatoid factor and C3/C4, all of which were negative. Antibodies against intrinsic factor were also found to be negative.

The patient received four packed red blood cells during the hospital stay and never received any plasmapheresis. As the patient was on high-dose metformin for many years and had no obvious other etiology to explain the cobalamin deficiency, we suspected that the patient’s symptoms and lab findings could be due to cobalamin deficiency from metformin use. We discontinued metformin and prescribed intramuscular (IM) cobalamin 1,000 μg daily for one week followed by the same dose IM cobalamin weekly for one month as the initial therapy. We also recommended upper gastrointestinal endoscopy to conclusively rule out pernicious anemia but the patient refused. Therefore, later we prescribed IM cobalamin 1,000 μg monthly life long. The patient improved clinically thereafter, and lab work at six-month follow-up is given in Table 1. We do plan to consider a trial without cobalamin in the future to see how the patient responds.

**Table 1:** Laboratory investigations WBC, white blood cell; MCV, mean corpuscular volume; RDW, red cell distribution width; APTT, partial thromboplastin time; BUN, blood urea nitrogen; AST,aspartate aminotransferase; ALT, alanine transaminase; LDH, lactate dehydrogenase; TIBC, total iron-binding capacity; N/A, not available.

	Reference range	Admission	Discharge	Six-month f/u
Complete blood count				
WBC count	4.0-10.8 x 10^3^/uL	2.4	3.5	8.6
Hemoglobin	11.5-16.0 g/dL	3.7	8.0	11.9
Hematocrit	34.0%-47.0%	10.8	24.0	37
MCV	81.0-99.0 FL	95.6	92	77
RDW	11.5%-15.0%	34.9	25.9	15.7
Platelet count	140-400 x 10^3^/uL	94	72	390
Reticulocyte count number	28.6-139.0 x 10^3^/uL	19.3	326	N/A
Coagulation studies				
Prothrombin time	9.0-12.0 seconds	11.2	N/A	N/A
APTT	22.0-34.0 seconds	23.6	N/A	N/A
Fibrinogen	160-400 mg/dL	312	N/A	N/A
Chemistry				
BUN	8-20 mg/dL	22	14	12
Creatinine	0.5-0.9 mg/dL	1.2	1.0	0.97
Total bilirubin	0.3-1.2 mg/dL	1.4	N/A	0.6
AST	7-37 U/L	113	N/A	52
ALT	10-49 U/L	50	N/A	70
LDH	120-246 U/L	4320	2360	N/A
Iron	35-160 ug/dL	88	N/A	N/A
TIBC	250-400 mcg/dL	334	N/A	N/A
Iron saturation	15%-50%	26	N/A	N/A
Ferritin	10-291 mcg/L	79	N/A	N/A
Vitamin B12	220-1000 pg/mL	91	>2000	N/A
Folate	3.0-16.0 ng/mL	16.6	N/A	N/A
Haptoglobin	40-240 mg/dL	<1	N/A	N/A
Methylmalonic acid	≤0.4 nmol/mL	31	N/A	N/A
Intrinsic factor antibody	Negative	Negative	N/A	N/A

## Discussion

Even though cobalamin deficiency is common in the general population, its hematologic manifestations, which resemble TTP, are seen in only 2.5% of the cobalamin-deficient patients [2]. The association between metformin and cobalamin deficiency has long been described since 1969 [3]. The first case of MICD causing megaloblastic anemia was published in 1980 [4]. However, according to our literature search no case of MICD causing pseudo-TTP has been reported.

The mechanism for MICD has been controversial. The proposed theories have included inhibition of cobalamin absorption, changes in intrinsic factor levels, normal flora or gastrointestinal (GI) motility [5]. The cubulin endocytic receptor formed by the 460-kDa protein cubilin and the 45-kDa transmembrane protein amnionless, is responsible for the absorption of cobalamin-intrinsic factor complex in the intestine [6]. Metformin has been found to disrupt the calcium-dependent membrane activity of this receptor, and hence decreases the absorption of cobalamin by the body [5]. Decreased serum cobalamin levels can be seen as early as three to four months after starting metformi;, however, symptomatic deficiency takes 5-10 years [5]. The risk for developing MICD increases with age, higher dose and longer duration of treatment.

The pathogenesis of cobalamin deficiency causing pseudo-TTP is also not completely understood. Cobalamin deficiency cannot only lead to ineffective erythropoiesis but can also increase red blood cell rigidity, which can in turn promote intramedullary destruction of red cell precursors [7]. As the erythrocyte precursor cells are nucleated and contain very little hemoglobin, lysis of these cells translates into laboratory findings of very high LDH (>2,500 IU/L), reticulocytopenia and relatively normal bilirubin which can be used to distinguish from TTP [1,8]. Additionally, cobalamin deficiency can increase homocysteine levels in the blood, which is associated with endothelial injury, vasoconstriction, stimulation of the coagulation cascade and increased platelet aggregation, all of which can promote intravascular hemolysis [7]. This would be reflected by the presence of schistocytes (Figure 1) and bizarre anisopoikilocytosis on the peripheral blood smear (Figure 2). These abnormal blood findings can affect the mean corpuscular volume; however, it has been found to be normal in less than 50% of the patients [9]. Moreover, D-dimer levels can also be elevated in some patients due to activation of the coagulation cascade and co-relate well with the degree of schistocytosis [2].

**Figure 3:**
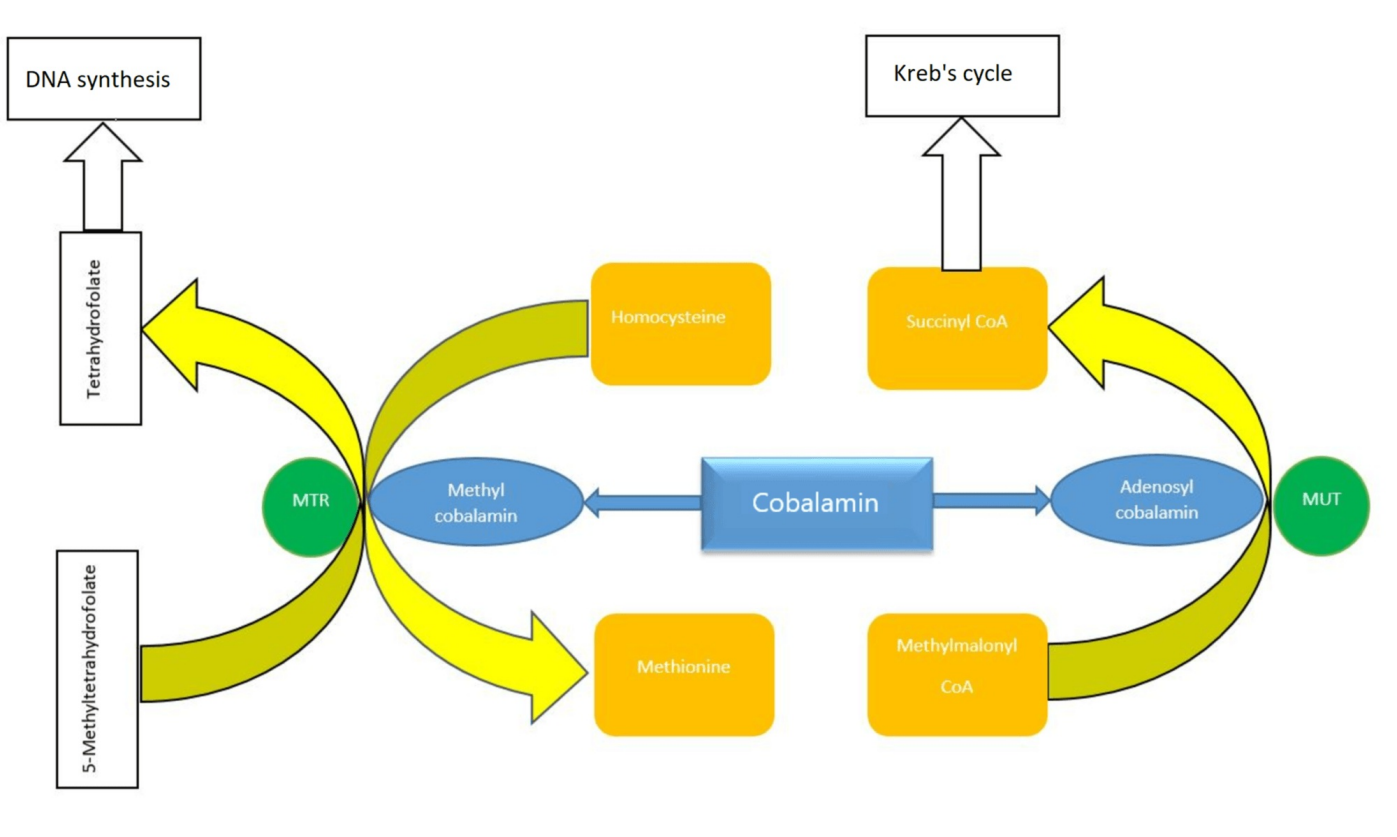
Diagram showing cobalamin involvement in DNA synthesis MTR- 5, methyltetrahydrofolate-homocysteine methyltransferase; MUT, methylmalonyl coenzyme A mutase.

Cobalamin is necessary for DNA synthesis and hematopoiesis (Figure 3). Cobalamin deficiency cannot only cause megaloblastic anemia but also thrombocytopenia and leukopenia, which is consistent with our findings. The degree of thrombocytopenia can distinguish between pseudo-TTP and TTP. The average platelet count seen with pseudo-TTP is approximately 70 x 10^3^/μL, whereas the average platelet count associated with TTP is approximately 12.5 x 10^3^/μL [10]. Hepatosplenomegaly has been seen with some cases of severe cobalamin deficiency because of increased extramedullary hematopoiesis in response to the severe anemia [11].

Although ADAMTS13 is usually normal in cobalamin deficiency, some case reports have reported ADAMTS13 to be surprisingly low [7]. This can be explained by the fact that a lack of cobalamin can lead to generation of oxidative stress, which can in turn elevate inflammatory markers such as interleukin 6 (IL-6), which suppress ADAMTS13 activity [7]. Furthermore, acute renal failure and neurological symptoms can sometimes be seen with pseudo-TTP but are generally more consistent with TTP [12].

There is no indication for plasmapheresis in the management of pseudo-TTP. Treatment varies depending on the underlying cause. Treatment initially involves IM cobalamin with 1,000 μg daily for one week, followed by 1,000 μg weekly for four weeks. We recommended monthly life-long supplementation in this particular case as patient refused upper GI endoscopy; hence, pernicious anemia could not be completely ruled out. According to the prior literature, reticulocyte count starts trending upwards by day 3 of treatment and peaks at one week. The hemoglobin concentration starts going up within 10 days and reaches normal limits by eight weeks [5]. These findings were consistent with our study.

## Conclusions

Pseudo-TTP can prove to be a diagnostic challenge and may incorrectly be treated with plasmapheresis which itself carries a high mortality rate. A carefully taken history and review of the medication list can help to avoid unnecessary interventions. It is important for clinicians to be aware of the link between metformin use and pseudo-TTP. Ordering simple bloods test like serum cobalamin level in a patient presenting with hemolysis and taking metformin as seen in our case can help to reach a timely diagnosis.
